# Investigating the Connection Between Endogenous Heme Accumulation and COX2 Activity in Cancer Cells

**DOI:** 10.3389/fonc.2019.00162

**Published:** 2019-03-19

**Authors:** Francesca Destefanis, Veronica Fiorito, Fiorella Altruda, Emanuela Tolosano

**Affiliations:** Department of Molecular Biotechnology and Health Sciences, Molecular Biotechnology Center, University of Torino, Torino, Italy

**Keywords:** COX2, FLVCR1, FLVCR, ALA, heme, haem, cancer, hemoprotein

## Abstract

Heme, an iron-containing porphyrin, is fundamental for a variety of functions in cell homeostasis. Nevertheless, recent data indicate that dysregulation of heme metabolism might promote tumorigenesis. The intracellular heme pool is finely regulated through the control of heme synthesis, degradation, incorporation into hemoproteins and trafficking across membranes. All these processes might be potentially targeted to alter endogenous heme content in order to counteract cancer growth. Nevertheless, these putative therapeutic interventions have to take into account the possibility of undesired side effects, such as the over-activation of heme-dependent enzymes involved in cancer. Among them, cyclooxygenase-2 is a prostaglandin-producing hemoprotein, induced during inflammation and in different types of tumor, particularly in colorectal cancer. The aim of this study was to evaluate whether modulation of endogenous heme may affect cyclooxygenase-2 expression and activity, taking advantage of two different approaches able to alter heme levels: the silencing of the heme exporter Feline Leukemia Virus subgroup C receptor 1 and the induction of heme synthesis by 5-aminolevulinic acid administration. Our data demonstrate that the down-regulation of the heme exporter in colorectal cancer cells does not affect cyclooxygenase-2 expression and activity. Conversely, 5-aminolevulinic acid administration results in decreased cyclooxygenase-2 expression. However, the overall cyclooxygenase-2 enzymatic activity is maintained. The present work sheds light on the complex modulation of cyclooxygenase-2 by endogenous heme and support the idea that targeting heme metabolism could be a valuable therapeutic option against cancer.

## Introduction

Heme is an iron-containing porphyrin that exerts a variety of functions in cell homeostasis. Despite its positive properties, excessive heme accumulation is often associated to cytotoxic effects. Therefore, a tight regulation of the intracellular heme pool is necessary to favor heme-dependent processes and, at the same time, to limit heme cytotoxicity. The alteration of this fine balance leads to cell death (1–5).

Targeting heme metabolism is emerging as a new therapeutic option to counteract tumor growth. In particular, the inactivation of the heme-degrading enzyme heme oxygenase-1 (HMOX1) is lethal in the context of hereditary leiomyomatosis and renal-cell cancer (HLRCC), a form of tumor characterized by germline mutations in the gene encoding for the enzyme fumarate hydratase (6). Moreover, in pediatric acute myeloid leukemia, high MYCN-expressing leukemic cells are dependent on porphyrin/heme export for their growth (7). In addition, suppression of the plasma membrane heme exporter Feline Leukemia Virus subgroup C receptor 1 (FLVCR1), which has a crucial role in the maintenance of heme homeostasis in several cell types (8–14), promotes autophagy and inhibition of tumor growth in synovial sarcoma cells (15). Finally, FLVCR1 expression is related to survival, disease status and prognosis in patients affected by hepatocellular carcinoma (16). All these data suggest that the accumulation of endogenous heme may be detrimental for tumor growth.

Heme homeostasis is maintained by a balance among heme synthesis, incorporation into hemoproteins, degradation and trafficking across cell membranes (1). In theory, all these processes might be potential targets to alter endogenous heme content in order to counteract tumor growth. Nevertheless, appropriate intracellular heme levels are crucial to modulate proper hemoproteins functions. Thus, putative therapeutic interventions targeting heme metabolism have to consider the possibility that the modulation of endogenous heme might result in unintended side effects, including the undesired over-activation of heme-dependent enzymes with a recognized role in cancer.

In a previous work (17) we have clarified a complex heme-dependent modulation of the hemoproteins cytochromes P450, showing that their enzymatic activity is controlled by newly synthesized heme rather than by the intracellular heme amount. In the present work we focus our analysis on cyclooxygenase-2 (COX2), a prostaglandins producing hemoprotein. This enzyme has a particularly significant role for cancer cells, being specifically overexpressed in several different types of cancer (18–23). The production of prostaglandins, particularly prostaglandin E_2_, by COX2 has been demonstrated to affect several aspects of tumor growth and progression, including tumor cell proliferation, migration, invasion, tumor angiogenesis, escape to tumor immunosurveillance (24), so COX2 appears to have a prominent role in cancer compared to other hemoproteins. The importance of COX2 for cancer is underlined by the strong interest in the research of anti-tumor compound targeting this enzyme (25–28).

Previous studies showed that heme acts as a cofactor for COX2, promoting its correct function (29, 30). Moreover, exogenous heme has been demonstrated to contribute to the control of COX2 activity (31–33).

The aim of the present study was to determine whether modulation of endogenous heme may affect COX2 expression and activity.

Since COX2 is overexpressed in approximately 80% of colorectal cancers (CRCs) (20), we chose CRC as a model and we took advantage of two different approaches, both able to modulate the intracellular heme content. The first strategy was the suppression of the heme exporter FLVCR1a to block cellular heme export and promote its accumulation in the cytosol. The second approach was cell treatment with 5-aminolevulinic acid (ALA), the heme precursor (34), in order to boost heme biosynthesis.

## Materials and Methods

### Cell Culture

SNU407 (KCLB, ID 407) and HCA-24 cells (ECACC, ID 6061903) were propagated in RPMI medium (Thermo Fisher Scientific) and DMEM medium (Thermo Fisher Scientific), respectively, supplemented with 10% heat-inactivated low-endotoxin fetal bovine serum (FBS) and 2 mM L-glutamine (Thermo Fisher Scientific). All cell media were ordinarily supplemented with antibiotics (100 U/ml penicillin and 100 μg/ml streptomycin; Thermo Fisher Scientific). Cells were maintained in a 37°C and 5% CO_2_ air incubator and routinely screened for absence of Mycoplasma contamination.

### RNA Extraction and Quantitative Real-Time PCR Analysis

RNA extraction and quantitative real-time PCR analysis were performed as described previously (35).

Briefly, total RNA was extracted using Purelink micro to midi RNA extraction kit (Invitrogen, San Giuliano Milanese, Italy). Between 500 and 1,000 ng of total RNA were transcribed into complementary DNA (cDNA) by M-MLV reverse transcriptase and random primers (Invitrogen, San Giuliano Milanese, Italy). Quantitative real-time PCR (qRT-PCR) was performed using the Universal Probe Library system (Roche). Primers and probes were designed using the ProbeFinder software (http://www.roche-applied-science.com). For *FLVCR1a*, specific primers and the probe were designed using Primer Express Software Version 3.0 (Applied Biosystem). qRT-PCR were performed on a 7900 Real Time PCR System (Applied Biosystems, Monza, Italy) and the analyses were done using RQ Manager software. Transcript abundance, normalized to beta-actin mRNA expression, is expressed as a fold increase over a calibrator sample.

### 5-Aminolevulinic Acid Treatment

To enhance heme biosynthesis, cells were treated with 5 mM 5-aminolevulinic acid (ALA, A3785; Sigma-Aldrich) for 5 and 24 h, as reported in Fiorito et al. (8), Petrillo et al. (9), Chiabrando et al. (11), Castori et al. (13), Vinchi et al. (17), and Chiabrando et al. (36).

### Western Blot

Western blot analysis was performed on total cell lysates according to standard procedures using antibodies against COX2 (Abcam, Cambridge, UK, ab15191, diluted 1:500), ALAS1 (Abcam, Cambridge, UK, ab84962, diluted 1:1,000), HMOX1 (Enzo Life Sciences, Farmingdale, NY, ADI-SPA-896, diluted 1:300), actin (Santa Cruz Biotechnology Inc., Santa Cruz, California, USA, I-19, diluted 1:1,000) and vinculin (homemade mouse monoclonal antibody, diluted 1:8,000).

### FLVCR1a Silencing

For gene silencing, a shRNA (TRC Lentiviral pLKO.1 Human FLVCR1 shRNA set RHS4533-NM_014053, clone TRCN0000059599; Thermo Fisher Scientific, Inc.) targeting the first exon of the human *FLVCR1* gene was used to specifically down-regulate *FLVCR1a*. For control cells, a pLKO.1 scramble shRNA was used. Following lentiviral transduction, cells were maintained in selective medium containing 0.02 μg/ml puromycin.

### Measurement of Heme Concentration

Intracellular heme concentration was measured using a fluorescence assay (8, 17, 37–40). Briefly, cells untreated or treated with 5 mM ALA for 5 and 24 h were collected and 2M oxalic acid was added. Samples were then heated at 95°C for 30 min leading to iron removal from heme. Fluorescence (wavelength: excitation 400—emission 662 nm) of the resultant protoporphyrin was assessed on a Glomax Multi Detection System (Promega Corporation). The endogenous protoporphyrin content (measured in parallel unheated samples in oxalic acid) was subtracted. Data were normalized to total protein concentration in each sample. Results are expressed as pmol of heme/mg total protein.

### COX2 Activity Assay

COX2 activity was assessed using a fluorometric kit provided by Abcam, Cambridge, UK (ab204699). Fluorescence emitted by the probe during the reaction (wavelength: excitation 535 nm - emission 587 nm) was assessed on a Glomax Multi Detection System (Promega Corporation). Results were expressed as pmol/min.mg.

### Statistical Analyses

Results are expressed as mean ± SEM. Statistical analyses were performed using one-way or two-way analysis of variance (ANOVA), followed by Bonferroni correction, for multiple group comparisons. An unpaired Student's *t*-test was used when only two groups were compared. A *p* < 0.05 was regarded as significant.

## Results

### FLVCR1a Suppression in SNU407 Cells Does Not Alter COX2 Expression and Activity

Previous data indicated that suppression of the plasma membrane heme exporter FLVCR1a is often associated to intracellular heme accumulation (8, 9). Thus, to investigate the possible correlation between heme metabolism and COX2 expression and activity, we silenced *FLVCR1a* gene using a specific shRNA in SNU407 and HCA-24 cell lines, characterized by high FLVCR1a and COX2 expression (data not shown).

**Figure 1:**
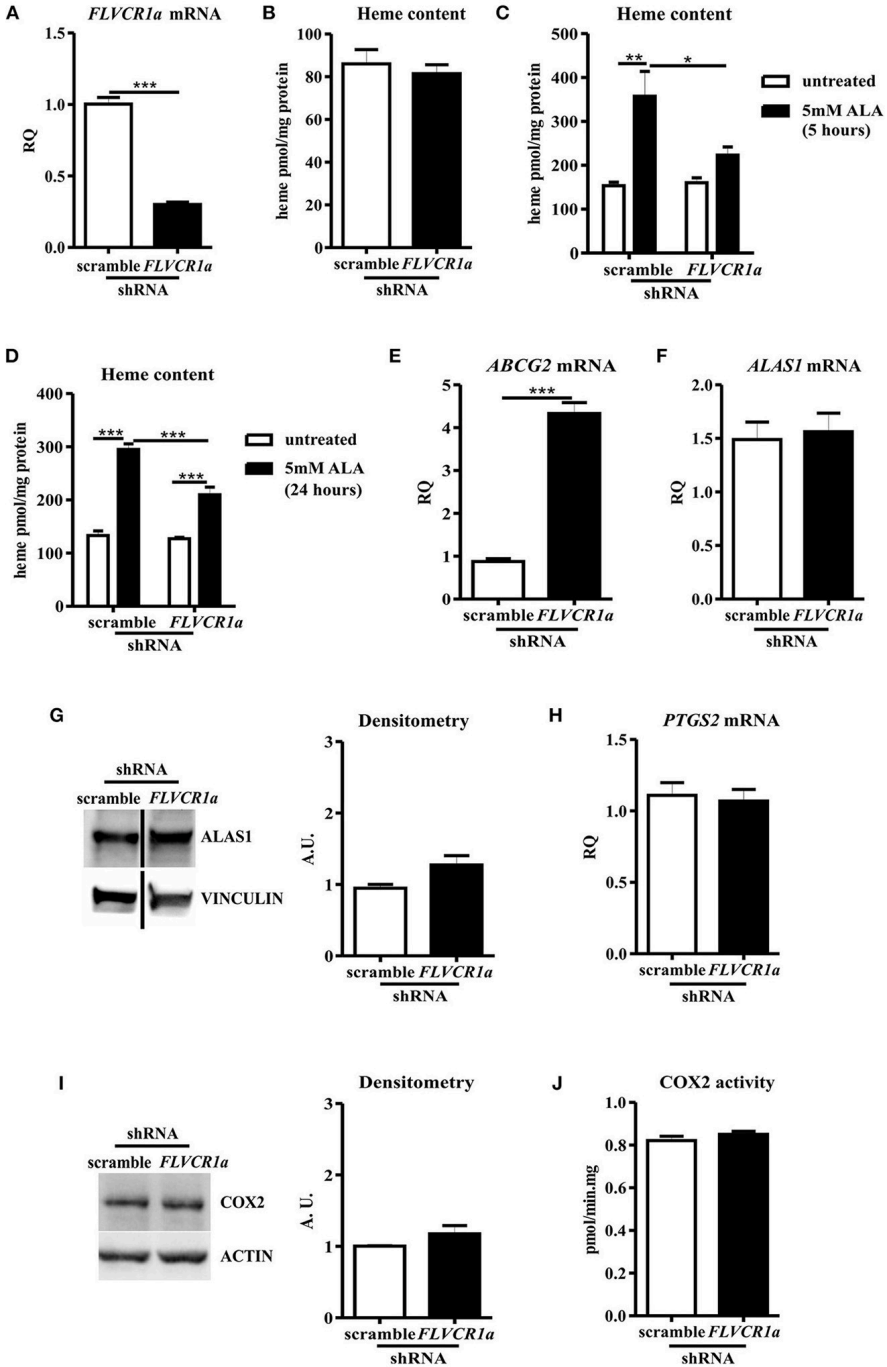
FLVCR1a suppression in SNU407 cells does not alter COX2 expression and activity. **(A)** qRT-PCR analysis of *FLVCR1a* expression in SNU407 cells, in which the expression of *FLVCR1a* was downregulated using a specific shRNA. Transcript abundance, normalized to beta-actin mRNA expression, is expressed as a fold increase over a calibrator sample (RQ = Relative Quantity). Data represent mean ± SEM, *n* = 6; ***p < 0.001. **(B)** Heme content in SNU407 cells, in which the expression of *FLVCR1a* was downregulated using a specific shRNA. Values are expressed as pmol/mg protein. Data represent mean ± SEM, *n* = 6. **(C,D)** Heme content in ALA-treated SNU407 cells, in which the expression of *FLVCR1a* was downregulated using a specific shRNA. Cells were treated with 5 mM ALA for 5 h **(C)** and 24 h **(D)**. Values are expressed as pmol/mg protein. Data represent mean ± SEM, *n* = 3; *p < 0.05, **p < 0.01, ***p < 0.001. **(E)** qRT-PCR analysis of *ABCG2* expression in SNU407 cells, in which the expression of *FLVCR1a* was downregulated using a specific shRNA. Transcript abundance, normalized to beta-actin mRNA expression, is expressed as a fold increase over a calibrator sample (RQ = Relative Quantity). Data represent mean ± SEM, *n* = 3; ***p < 0.001. **(F)** qRT-PCR analysis of *ALAS1* expression in SNU407 cells, in which the expression of *FLVCR1a* was downregulated using a specific shRNA. Transcript abundance, normalized to beta-actin mRNA expression, is expressed as a fold increase over a calibrator sample (RQ = Relative Quantity). Data represent mean ± SEM, *n* = 6. **(G)** Representative Western blot of ALAS1 expression in *FLVCR1a*-silenced SNU407 cells. Band intensities were measured by densitometry and normalized to vinculin expression (A. U. = Arbitrary Unit). Densitometry data represent mean ± SEM, *n* = 2. **(H)** qRT-PCR analysis of *PTGS2* expression in *FLVCR1a*-silenced SNU407 cells. Transcript abundance, normalized to beta-actin mRNA expression, is expressed as a fold increase over a calibrator sample (RQ = Relative Quantity). Data represent mean ± SEM, *n* = 6. **(I)** Representative Western blot of COX2 expression in *FLVCR1a*-silenced SNU407 cells. Band intensities were measured by densitometry and normalized to actin expression (A. U. = Arbitrary Unit). Densitometry data represent mean ± SEM, *n* = 2. **(J)** COX2 activity in SNU407 cells, in which the expression of *FLVCR1a* was downregulated using a specific shRNA. Values are expressed as pmol/min.mg protein. Data represent mean ± SEM, *n* = 2.

Once confirmed *FLVCR1a* down-regulation in SNU407 cells (Figure 1A), we checked for the intracellular heme amount. Unexpectedly, heme did not accumulate in *FLVCR1a*-silenced cells, as compared to control cells (Figure 1B), indicating possible cell compensatory mechanisms to deal with FLVCR1a loss.

To further examine this point, we analyzed the rate of heme accumulation in the time by treating SNU407 cells with the heme precursor ALA. A lower amount of heme was accumulated 5 and 24 h after ALA administration in *FLVCR1a*-silenced cells as compared to controls (Figures 1C,D), indicating a slower rate of porphyrin synthesis or a faster system of heme export/degradation. Supporting the latter idea, *FLVCR1a*-silenced cells clearly showed the up-regulation of the mRNA for *ABCG2* (ATP-Binding Cassette, subfamily G, member 2) (Figure 1E), a plasma membrane transporter which can function as a porphyrins exporter (41, 42). Conversely, *FLVCR1a* silencing did not affect both 5-aminolevulinic acid synthase-1 (ALAS1) mRNA and protein levels in SNU407 cells (Figures 1F,G), although alterations in ALAS1 activity cannot be excluded.

Anyhow, the inability to alter heme content by FLVCR1a depletion in SNU407 cells led to negligible modulation of COX2 mRNA and protein levels (Figures 1H,I). Moreover, COX2 enzyme activity was unaffected (Figure 1J).

Similar results were obtained in HCA-24 cells (Supplemental Figure 1).

### ALA Treatment Decreased COX2 Protein Levels, With Negligible Effects on the Overall Enzyme Activity

**Figure 2:**
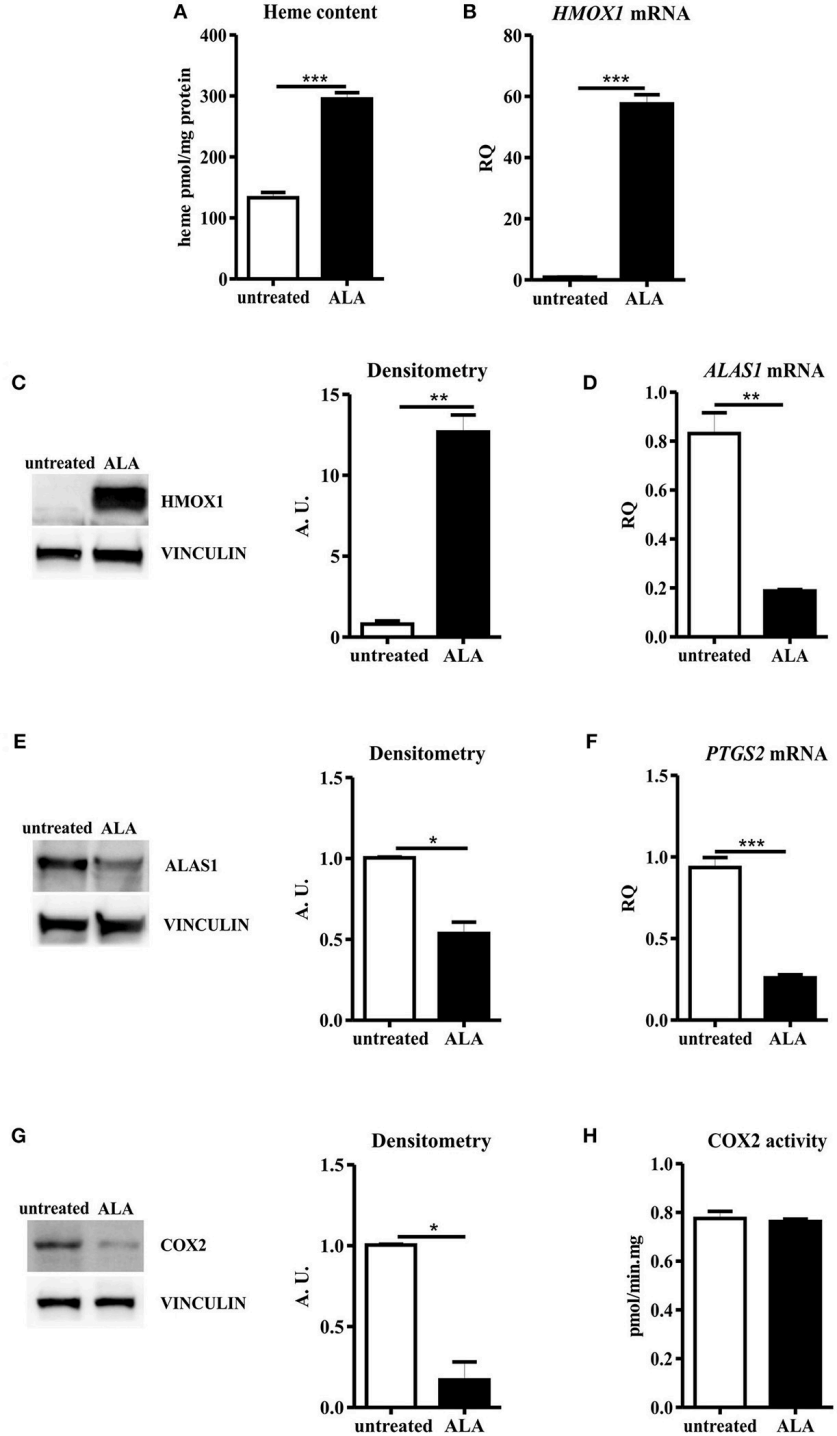
ALA treatment decreased COX2 protein levels, with negligible effects on the overall enzyme activity. **(A)** Heme content in SNU407 cells untreated or treated with 5 mM ALA for 24 h. Values are expressed as pmol/mg protein. Data represent mean ± SEM, *n* = 3; ***p < 0.001. **(B)** qRT-PCR analysis of *HMOX1* expression in SNU407 cells untreated or treated with 5 mM ALA for 24 h. Transcript abundance, normalized to beta-actin mRNA expression, is expressed as a fold increase over a calibrator sample (RQ = Relative Quantity). Data represent mean ± SEM, *n* = 3; ***p < 0.001. **(C)** Representative Western blot of HMOX1 expression in SNU407 cells untreated or treated with 5 mM ALA for 24 h. Band intensities were measured by densitometry and normalized to vinculin expression (A. U. = Arbitrary Unit). Densitometry data represent mean ± SEM, *n* = 2; **p < 0.01. **(D)** qRT-PCR analysis of *ALAS1* expression in SNU407 cells untreated or treated with 5 mM ALA for 24 h. Transcript abundance, normalized to beta-actin mRNA expression, is expressed as a fold increase over a calibrator sample (RQ = Relative Quantity). Data represent mean ± SEM, *n* = 3; **p < 0.01. **(E)** Representative Western blot of ALAS1 expression in SNU407 cells untreated or treated with 5 mM ALA for 24 h. Band intensities were measured by densitometry and normalized to vinculin expression (A. U. = arbitrary unit). Densitometry data represent mean ± SEM, *n* = 2; *p < 0.05. **(F)** qRT-PCR analysis of *PTGS2* expression in SNU407 cells untreated or treated with 5 mM ALA for 24 h. Transcript abundance, normalized to beta-actin mRNA expression, is expressed as a fold increase over a calibrator sample (RQ = Relative Quantity). Data represent mean ± SEM, *n* = 3; ***p < 0.001. **(G)** Representative Western blot of COX2 expression in SNU407 cells untreated or treated with 5 mM ALA for 24 h. Band intensities were measured by densitometry and normalized to vinculin expression (A. U. = arbitrary unit). Densitometry data represent mean ± SEM, *n* = 2; *p < 0.05. **(H)** COX2 activity in SNU407 cells untreated or treated with 5 mM ALA for 24 h. Values are expressed as pmol/min.mg protein. Data represent mean ± SEM, *n* = 4.

ALA treatment is a proved strategy to boost heme biosynthesis, leading to increased intracellular heme amount and modulation of heme metabolism (8, 9, 17). As expected, we observed an increase in intracellular heme content in SNU407 cells upon ALA administration (Figure 2A). As a consequence, upregulation of HMOX1 and down-regulation of ALAS1 occurred in treated cells, both at transcriptional and post-transcriptional levels (Figures 2B–E). Collectively, these data indicate that ALA is a more potent stimulus than FLVCR1a suppression to promote heme accumulation.

ALA promotion of cellular heme biosynthesis has been demonstrated to efficaciously boost the activity of hemoproteins such as cytochromes P450 (17), likely providing newly synthesized heme to favor the turnover of the prosthetic group in the enzyme. Moreover, ALA administration leads to increased HMOX1 expression. Heme degradation by HMOX1 provides carbon monoxide (CO), a recognized inhibitor of COX2 gene (*PTGS2*) transcription (43). Therefore, upon ALA treatment, a complex COX2 modulation could occur. To dissect this point, we determined COX2 expression levels in ALA-treated SNU407 cells: real-time PCR and Western blot analyses showed decreased COX2 mRNA and protein levels in treated cells as compared to the untreated ones (Figures 2F,G). Nevertheless, when COX2 function was assessed, we observed a comparable total COX2 activity in untreated and ALA-treated cells (Figure 2H). Similar results were obtained in HCA-24 cells (Supplemental Figure 2).

Thus, ALA leads to decreased COX2 protein production, but the overall COX2 activity is maintained.

Collectively, these results indicate that, upon ALA treatment, despite reduced COX2 protein levels, cells can likely boost the function of the remaining COX2 molecules to maintain constant the overall COX2 activity.

## Discussion

The aim of this project was to evaluate whether modulations of endogenous heme could alter COX2 expression and activity in CRC cell lines.

According to the data collected, slight alterations in the amount of intracellular heme, obtained by blocking FLVCR1a-mediated heme efflux, do not affect COX2 expression and activity, indicating that cells can tolerate little modulation of intracellular heme without compromising the function of hemoproteins like COX2. This system appears different from those regulating cytochromes, where the block of FLVCR1a-mediated heme export is sufficient to alter their activity (17).

The promotion of heme synthesis by ALA administration, conversely, is able to modulate COX2 expression, leading to decreased protein levels. Several mechanisms control COX2 levels (44) and could potentially account for this down-modulation. However, in the experimental context reported herein, the reduction of COX2 protein synthesis could likely be ascribed to HMOX1. Indeed, ALA-mediated stimulation of heme synthesis leads to HMOX1 up-regulation, and HMOX1 has been reported to act as a potent negative regulator of COX2 protein expression by CO-mediated inhibition of *PTGS2* gene transcription (43). Interestingly, the down-modulation of COX2 protein upon ALA treatment does not reduce total COX2 activity. This means that ALA-induced heme synthesis provides the amount of new heme necessary to boost the activity of the residual COX2 protein, in order to maintain the total enzymatic activity. The fact that the synthesis of new heme can sustain the activity of an hemoprotein, likely by favoring the turnover of its prosthetic group, has already been reported for other heme-dependent enzymes like cytochromes P450 (17, 45, 46). In other words, increasing heme synthesis by ALA on one hand produces heme that stimulates COX2 activity, but on the other hand induces HMOX1 that decreases COX2 expression. The net effect is that the overall cellular COX2 activity is maintained constant.

We do not know the biological significance for the fact that heme can act as both an inducer and an inhibitor of COX2. It could be that this is a fine mechanism to control COX2 cellular activity. Moreover, we can speculate that cells developed a physiological system able to cope modulations of heme metabolism without interfering with the total COX2 activity in order to avoid inappropriate increase of COX2 activity in response to possible frequent fluctuations of intracellular heme synthesis.

Anyhow, the data reported collectively indicate that the sole modulation of heme synthesis/export is not sufficient to efficiently affect the overall COX2 cellular activity.

The present work shed light on the complex modulation of COX2 by endogenous heme at physiological levels. This might be relevant in the context of cancer therapy. Indeed, the alteration of heme metabolism represents an emerging strategy to counteract tumor growth, but heme targeting is still debated due to the risk of favoring the activity and expression of hemoproteins with a recognized role in cancer. The data reported herein indicate that, at least for COX2, tumor cells can tolerate changes in endogenous heme levels without altering the overall enzymatic activity, thus reinforcing the idea of targeting heme metabolism for therapeutic purposes.

## Author Contributions

FD designed and performed the experiments, made the analyses, and wrote the paper. FA was involved in study design. VF and ET designed the experiments, analyzed the data, and wrote the paper.

### Conflict of Interest Statement

The authors declare that the research was conducted in the absence of any commercial or financial relationships that could be construed as a potential conflict of interest.

## Supplementary Material

Supplemental Figure 1 FLVCR1a suppression in HCA-24 cells does not alter COX2 expression and activity. **(A)** qRT-PCR analysis of FLVCR1a expression in HCA-24 cells in which the expression of FLVCR1a was downregulated using a specific shRNA. Transcript abundance, normalized to beta-actin mRNA expression, is expressed as a fold increase over a calibrator sample (RQ = Relative Quantity). Data represent mean ± SEM, *n* = 6; ^***^*p* < 0.001. **(B)** Heme content in HCA-24 cells in which the expression of FLVCR1a was downregulated using a specific shRNA. Values are expressed as pmol/mg protein. Data represent mean ± SEM, *n* = 6. **(C)** Heme content in FLVCR1a-silenced HCA-24 cells untreated or treated with 5mM ALA for 24 h. Values are expressed as pmol/mg protein. Data represent mean ± SEM, *n* = 3; ^**^*p* < 0.01. **(D)** qRT-PCR analysis of ALAS1 expression in HCA-24 cells, in which the expression of FLVCR1a was downregulated using a specific shRNA. Transcript abundance, normalized to beta-actin mRNA expression, is expressed as a fold increase over a calibrator sample (RQ = Relative Quantity). Data represent mean ± SEM, *n* = 6. **(E)** qRT-PCR analysis of PTGS2 expression in HCA-24 cells, in which the expression of FLVCR1a was downregulated using a specific shRNA. Transcript abundance, normalized to beta-actin mRNA expression, is expressed as a fold increase over a calibrator sample (RQ = Relative Quantity). Data represent mean ± SEM, *n* = 6. **(F)** Representative Western blot of COX2 expression in FLVCR1a-silenced HCA-24 cells. Band intensities were measured by densitometry and normalized to actin expression (A. U = Arbitrary Unit). Densitometry data represent mean ± SEM, *n* = 2.

Supplemental Figure 2 ALA treatment decreased COX2 protein levels, with negligible effects on the overall enzyme activity. **(A)** Heme content in HCA-24 cells untreated or treated with 5mM ALA for 24 h. Values are expressed as pmol/mg protein. Data represent mean ± SEM, *n* = 3; ^*^*p* < 0.05. **(B)** qRT-PCR analysis of HMOX1 expression in HCA-24 cells untreated or treated with 5mM ALA for 24 h. Transcript abundance, normalized to beta-actin mRNA expression, is expressed as a fold increase over a calibrator sample (RQ = Relative Quantity). Data represent mean ± SEM, *n* = 3; ^***^*p* < 0.001. **(C)** qRT-PCR analysis of ALAS1 expression in HCA-24 cells untreated or treated with 5mM ALA for 24 h. Transcript abundance, normalized to beta-actin mRNA expression, is expressed as a fold increase over a calibrator sample (RQ = Relative Quantity). Data represent mean ± SEM, *n* = 3; ^***^*p* < 0.001. **(D)** qRT-PCR analysis of PTGS2 expression in HCA-24 cells untreated or treated with 5mM ALA for 24 h. Transcript abundance, normalized to beta-actin mRNA expression, is expressed as a fold increase over a calibrator sample (RQ = Relative Quantity). Data represent mean ± SEM, *n* = 3. **(E)** Representative Western blot of COX2 expression in HCA-24 cells untreated or treated with 5 mM ALA for 24 h. Band intensities were measured by densitometry and normalized to vinculin expression (A. U. = Arbitrary Unit). Densitometry data represent mean ± SEM, *n* = 2; ^**^*p* < 0.01. **(F)** COX2 activity in HCA-24 cells untreated or treated with 5mM ALA for 24 h. Values are expressed as pmol/min.mg protein. Data represent mean ± SEM, *n* = 2.
