# Supplementary figures and images for: Investigating the Connection Between Endogenous Heme Accumulation and COX2 Activity in Cancer Cells
Source: Front Oncol. 2019 Mar 19;9:162. doi: 10.3389/fonc.2019.00162 (PMC6433962; doi:10.3389/fonc.2019.00162)

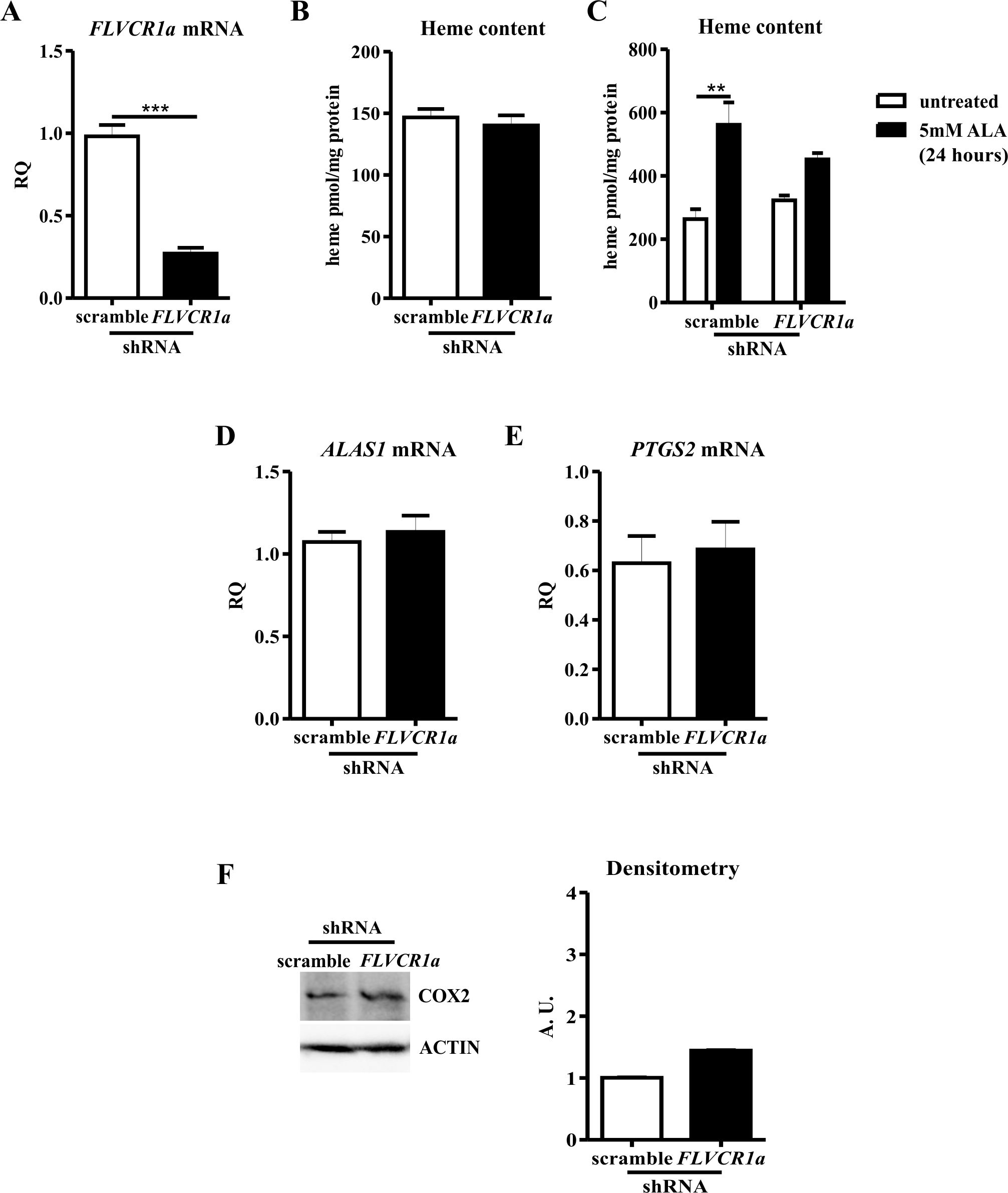

Supplement: Supplemental Figure 1 — FLVCR1a suppression in HCA-24 cells does not alter COX2 expression and activity. (A) qRT-PCR analysis of FLVCR1a expression in HCA-24 cells in which the expression of FLVCR1a was downregulated using a specific shRNA. Transcript abundance, normalized to beta-actin mRNA expression, is expressed as a fold increase over a calibrator sample (RQ = Relative Quantity). Data represent mean ± SEM, n = 6; ***p < 0.001. (B) Heme content in HCA-24 cells in which the expression of FLVCR1a was downregulated using a specific shRNA. Values are expressed as pmol/mg protein. Data represent mean ± SEM, n = 6. (C) Heme content in FLVCR1a-silenced HCA-24 cells untreated or treated with 5mM ALA for 24 h. Values are expressed as pmol/mg protein. Data represent mean ± SEM, n = 3; **p < 0.01. (D) qRT-PCR analysis of ALAS1 expression in HCA-24 cells, in which the expression of FLVCR1a was downregulated using a specific shRNA. Transcript abundance, normalized to beta-actin mRNA expression, is expressed as a fold increase over a calibrator sample (RQ = Relative Quantity). Data represent mean ± SEM, n = 6. (E) qRT-PCR analysis of PTGS2 expression in HCA-24 cells, in which the expression of FLVCR1a was downregulated using a specific shRNA. Transcript abundance, normalized to beta-actin mRNA expression, is expressed as a fold increase over a calibrator sample (RQ = Relative Quantity). Data represent mean ± SEM, n = 6. (F) Representative Western blot of COX2 expression in FLVCR1a-silenced HCA-24 cells. Band intensities were measured by densitometry and normalized to actin expression (A. U = Arbitrary Unit). Densitometry data represent mean ± SEM, n = 2. [file Image_1.JPEG]

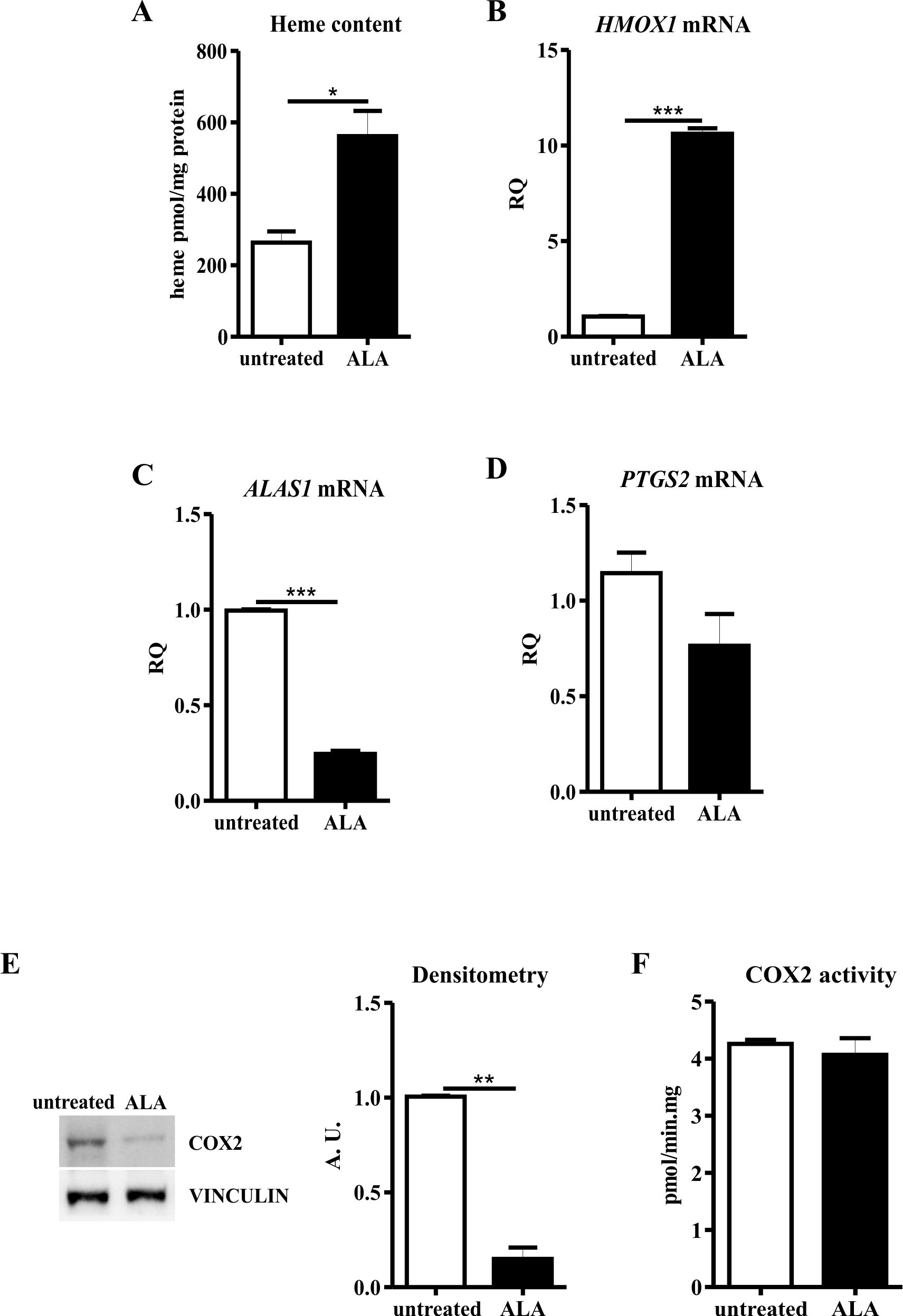

Supplement: Supplemental Figure 2 — ALA treatment decreased COX2 protein levels, with negligible effects on the overall enzyme activity. (A) Heme content in HCA-24 cells untreated or treated with 5mM ALA for 24 h. Values are expressed as pmol/mg protein. Data represent mean ± SEM, n = 3; *p < 0.05. (B) qRT-PCR analysis of HMOX1 expression in HCA-24 cells untreated or treated with 5mM ALA for 24 h. Transcript abundance, normalized to beta-actin mRNA expression, is expressed as a fold increase over a calibrator sample (RQ = Relative Quantity). Data represent mean ± SEM, n = 3; ***p < 0.001. (C) qRT-PCR analysis of ALAS1 expression in HCA-24 cells untreated or treated with 5mM ALA for 24 h. Transcript abundance, normalized to beta-actin mRNA expression, is expressed as a fold increase over a calibrator sample (RQ = Relative Quantity). Data represent mean ± SEM, n = 3; ***p < 0.001. (D) qRT-PCR analysis of PTGS2 expression in HCA-24 cells untreated or treated with 5mM ALA for 24 h. Transcript abundance, normalized to beta-actin mRNA expression, is expressed as a fold increase over a calibrator sample (RQ = Relative Quantity). Data represent mean ± SEM, n = 3. (E) Representative Western blot of COX2 expression in HCA-24 cells untreated or treated with 5 mM ALA for 24 h. Band intensities were measured by densitometry and normalized to vinculin expression (A. U. = Arbitrary Unit). Densitometry data represent mean ± SEM, n = 2; **p < 0.01. (F) COX2 activity in HCA-24 cells untreated or treated with 5mM ALA for 24 h. Values are expressed as pmol/min.mg protein. Data represent mean ± SEM, n = 2. [file Image_2.JPEG]
